# Counteracting Skin Aging In Vitro by Phytochemicals

**DOI:** 10.1111/jcmm.70530

**Published:** 2025-04-03

**Authors:** Sara Cruciani, Giuseppe Garroni, Diletta Serra, Fikriye Fulya Kavak, Rosanna Satta, Fernanda Martini, Mauro Tognon, Carlo Ventura, Margherita Maioli

**Affiliations:** ^1^ Department of Biomedical Sciences University of Sassari Sassari Italy; ^2^ Department of Medicine, Surgery and Pharmacy University of Sassari Sassari Italy; ^3^ Department of Medical Sciences University of Ferrara Ferrara Italy; ^4^ Laboratory of Molecular Biology and Stem Cell Engineering – Eldor Lab Istituto Nazionale Biostrutture e Biosistemi Bologna Italy; ^5^ Center for Developmental Biology and Reprogramming‐CEDEBIOR, Department of Biomedical Sciences University of Sassari Sassari Italy

**Keywords:** antioxidants, bioactive molecules, cell senescence, fibroblasts, gene expression, repairing mechanisms, skin aging, stem cells

## Abstract

The skin is the most extensive organ in the human body. Photo exposure to ultraviolet (UV) rays causes several damages to skin cells, including premature skin aging, the onset of possible DNA mutations, and the risk of developing cancers, including melanoma. Protecting skin from the damaging effects of sun exposure through the application of creams and filters is important to prevent irreversible damages. Several natural extracts and biomolecules with antioxidant activity are widely used in the production of dietary supplements or topical products, for the prevention and treatment of skin affections. Within this context, we pre‐treated human skin fibroblasts (HFF1), skin‐isolated stem cells (SSCs) and keratinocytes (HaCaT) with two creams containing a specific solar protection factor (SPF) for 72 h and then exposed the cells to UV light. Gene expression analysis was performed for the key cell cycle regulators (p16, p19, p21, p53 and TERT). Cell senescence was assessed by colorimetric assays of beta‐galactosidase and antioxidant potential, revealing the ability of treated cells to counteract free radical production as a result of oxidative stress. Finally, possible mutations in DNA induced by photo exposure were studied. The results obtained demonstrated that the tested products elicit positive effects on all skin cell populations, preserving them from photo exposure damages and premature senescence, being also able to increase the DNA repairing mechanisms and inducing a youngest phenotype.

## 1 Introduction

Skin is the most important barrier of our body, being involved in the maintenance of hydration, protection against pathogens, regulation of physiological temperature and vitamin D synthesis [1, 2]. Different cell populations may contribute to different skin layers, including stem cells (SCs), widely known for their participation in skin homeostasis [3]. Pressure or temperature modulates the cellular microenvironment, sensed by distinct cell populations and able to influence cell fate. Moreover, interactions between neighbouring cells in each skin layer are important regulators of cell behaviour [4]. Also, fibroblasts and keratinocytes participate in the maintenance of skin homeostasis, being the first actors in the inflammatory phase during reparative and regenerative processes [5, 6]. Excessive reactive oxygen species (ROS), imbalanced extracellular matrix (ECM) homeostasis or accumulation of senescent cells may cause systemic inflammation and accelerate age‐related dysfunction of the entire tissue [7, 8]. Several extrinsic factors can negatively impact cell behaviour and skin cell turnover. Alterations to the skin barrier integrity, as those seen following prolonged UV exposure, are associated with many skin conditions, including sunburn, photoaging, and skin cancer [9, 10]. The WHO report indicates the incidence of 300,000 non‐melanoma skin cancers due to chronic exposure to sunlight with a prevalence especially during adolescence, when exposure to harmful UV rays is common [11]. The damaging effects of continuous sun exposure can be divided into acute damage, represented by sunburn, erythema, painful edema, and photo dermatosis, and more severe chronic damage, such as photoaging and premalignant skin lesions [12]. Prolonged UVB and UVA irradiation generate severe oxidative stress in skin cells, with increased expression of activator protein 1 (AP‐1) activity and matrix metalloproteinases (MMPs), altered transforming growth factor β (TGF‐b) signalling, increased collagen and ECM degradation and increased inflammation [13]. Creams and other topical formulations based on natural molecules, containing a specific protective factor (SPF) are known to be effective against UV radiations, maintaining the right degree of skin hydration [14, 15]. No products currently on the market are capable of blocking 100% of the sun's rays, but in recent years, new formulations have been developed with a focus on improving UV protection, photostability and environmental sustainability [16]. Sunscreen formulations are usually alcohol‐based, lipophilic or a combination of both, capable of easily penetrating the stratum corneum [17]. Among the various combinations of sunscreens and active ingredients, oxybenzone, avobenzone, octisalate, octocrylene, homosalate, and octinoxate are generally used, but these are often found to be toxic and harmful to the skin when used for long periods [17]. Therefore, effective strategies to protect against UV‐induced skin damage are essential. Some newer sunscreens have therefore been designed to counteract oxidative stress damage by adding antioxidants or anti‐inflammatory molecules to the actives [18]. Aromatic plants, fruits and seeds contain molecules able to exert biological activity and are well known for their antioxidant, anti‐inflammatory, and antibacterial properties against some chronic diseases [19, 20]. Phytoplankton is used in cosmetics for different purposes and in a wide range of cosmetic preparations [21]. Phytoplankton is a group of microscopic plants that live in the ocean, in freshwater and in other terrestrial aqueous systems [22]. Due to in‐trace elements and the content of free amino acids, phytoplankton seems to be involved in restoring the activities of the proteasome, contributing to ROS scavenging activity and preventing the formation of oxidised proteins caused by UV radiation and aging [23, 24]. In addition, phytoplankton extract helps in restoring cell communication and skin barrier, reducing inflammation and restoring skin homeostasis after stress [25]. One of the best‐known UV radiation‐induced DNA damages is the formation of pyrimidine dimers, which are responsible for mutations and, in severe cases, tumours, interfering with both replication and transcription [26, 27]. To protect themselves from ultraviolet‐induced damage, cells have different DNA repairing mechanisms. Among these, certain enzymes are able to recognise a damage and correct it by harnessing light energy in a process known as photo‐reactivation [28, 29]. These enzymes, called photolyases, are present in many multicellular eukaryotic species but not in humans. Photolyases are the key enzymes that remove photodamages [30]. The activity of this enzyme occurs upon exposure to photo‐reactivating light, significantly reducing the apoptotic response of UV‐exposed skin cells [31]. Topical application of these enzymes within specially formulated sunscreens could contribute to enhancing damage repairing mechanisms and preventing UV‐induced tumour formation [32, 33]. In this regard, the aim of this study was to investigate the effects of two topical sunscreen products containing photolyase and phytoplankton extract on skin cell populations, such as fibroblasts, stem cells and keratinocytes, to evaluate their antioxidant effects, as well as preventive and repairing activities against DNA damages induced by UV exposure.

## 2 Materials and Methods

### 2.1 Materials

For in vitro tests, two topical products, one containing a SPF 25 UV solar filter and one containing a SPF 50+ UV solar filter, were used. The products were specifically formulated to contain a set of synergistic bioactive systems to reduce photo exposure damages and reconstitute skin, called DNA Reactivator Complex. This contains antioxidants, such as tocopherol and tocopheryl acetate, and molecules with shielding activity from UV‐A/UV‐B and blue light rays (polygonium avicular extract, *theobroma cacao* extract, bis‐ethylexyl hydroxydimethoxy benzylmalonate) and phytoplankton extract (photolyase), an enzyme that acts as a DNA repairing mechanism.

### 2.2 Cell Culturing Conditions

Human foreskin fibroblasts (HFF1) and human keratinocytes (HaCaT) were obtained from ATCC (Manassas, VA, USA) and cultured in a Dulbecco's modified Eagle's Medium (DMEM) (Life Technologies, Carlsbad, CA, USA), supplemented with 10% fetal bovine serum (FBS Life Technologies), 2 mM l‐glutamine (Euroclone, Milano, Italy) and 1% penicillin/streptomycin (Euroclone, Milano, Italy). Skin stem cells (SSCs) were isolated from skin biopsies of adult male and female patients, as previously described [34] after approval from the ethical committee (Ethical Clearance N. 0021565/2018, 22/03/2018‐Commissione Etica CNR). SSCs were then cultured in a Dulbecco's modified Eagle's Medium (DMEM) (Life Technologies, Carlsbad, CA, USA), supplemented with 15% fetal bovine serum (FBS, Life Technologies, Carlsbad, CA, USA), 2 mM l‐glutamine (Euroclone, Milano, Italy) and 1% penicillin/streptomycin (Euroclone, Milano, Italy).

Once reaching a confluence, each cell population (HaCaT, HFF1 and SSCs) was divided into different experimental groups: group (1) was used as a negative control and maintained in the culturing medium alone, without exposure to UV light; group (2) was used as a positive control of photo‐damages and exposed to UV light for 2 min at 10 cm distance from the UV lamp (253.7 nm, 30 W); group (3) was pre‐treated for 72 h with SPF25 cream and then exposed to UV light; group (4) was pre‐treated for 72 h with SPF50+ cream and then exposed to UV light.

For all experimental conditions, SPF25 and SPF50+ were dissolved in the growing medium.

### 2.3 Cytotoxicity Assay

Thiazolyl Blue Tetrazolium Bromide (MTT) assay (Sigma‐Aldrich, Germany) was used to evaluate the cytotoxicity of our product after 24, 48 and 72 h of treatment. Cells were seeded at a concentration of 8000 cells/well in 96‐well plates cultured in the presence or absence of creams, before exposure to UV light. The medium was then removed and 100 *μ*L of 0.65 mg/mL MTT was added in each well and incubated for 2 h. After incubation, the medium was removed, and formazan was dissolved in DMSO. The absorbance of each well was detected at 570 nm using a microplate reader (Akribis Scientific, Common Farm, Frog Ln, Knutsford WA16 0JG, Great Britain). The viability of cells pretreated with the creams was calculated as % cell viability referred to untreated control cells = (OD570 treated cells) × 100/ (OD570 control).

### 2.4 Cell Proliferation Assay

The BrdU assay (Cell Signalling Technology, Euroclone, Milan Italy) was used for the quantification of cell proliferation. HaCaT, HFF1 and SSCs were seeded in separate plates at a concentration of 8000 cells/well in 96‐well plates and treated under the above‐described conditions for 72 h. Cell viability was detected by measuring the absorbance of each well at 450 nm by plate reader (Akribis Scientific, Common Farm, Frog Ln, Knutsford WA16 0JG, Great Britain) and was expressed in OD units as compared to untreated control cells. Data were expressed as mean ± SD.

### 2.5 Gene Expression Analysis

Total RNA was extracted from HaCaT, HFF1 and SSCs cultured in the above‐described conditions using the RNeasy Mini Kit (Qiagen, 40,724 Hilden, Germany) after 72 h of culturing under the above‐described conditions. RNA was quantified by the NanoDrop One/OneC Microvolume UV–Vis spectrophotometer (Thermo Fisher Scientific, Grand Island, NY, USA) and approximately 1 μg of total RNA from each sample was amplified in a Real‐time quantitative PCR using the Luna Universal One‐Step RT‐qPCR Kit (New England Biolabs), using a CFX Thermal Cycler (Bio‐Rad, Hercules, CA, USA). Target Ct values of each sample were normalised to GAPDH, used as a reference gene. The relative values of all analysed genes were expressed as fold of change (2^−∆∆Ct^) of mRNA levels observed in untreated control cells. The primers used were from Thermo Fisher Scientific (Grand Island, NY, USA) and described in Table 1.

**TABLE 1:** Primer sequences.

Primer name	Forward	Reverse
GAPDH	GAGTCAACGGATTTGGTCGT	GACAAGCTTCCCGTTCTCAG
p16 INK4	CAACGCACCGCCTAGTTACGG	AACTTCGTCCTCCAGAGTCGC
p19 ARF	GCCTTCGGCTGACTGGCTGG	TCGTCCTCCAGAGTCGCCCG
p21	CAAAGGCCCGCTCTACATCTT	AGGAACCTCCATTCACCCGA
p53	CAAGCAATGGATGATTTGATGCT	TGGGTCTTCAGTGAACCATTGT
TERT	GACGTGGAAGATGAGCGTG	GACGACGTACACACTCATC
HSP70	CACAGCGACGTAGCAGCTCT	ATGTCGGTGGTGGGCATAGA

### 2.6 Antioxidant Capacity

To detect the total antioxidant capacity (TAC), the TAC Assay Kit (Abcam, Cambridge, UK) was used. Briefly, HaCaT, HFF1 and SSCs cultured in the above‐described conditions for 72 h were homogenised, and supernatants were collected as indicated in the manufacturer's instructions. 100 μL Cu^2+^ Working Solution was added to all standard and sample wells and incubated at room temperature for 90 min on an orbital shaker, protected from light. Absorbance of each sample was measured on a microplate reader (Akribis Scientific, Common Farm, Frog Ln, Knutsford WA16 0JG, Great Britain) at OD 570 nm. Trolox (6‐hydroxy‐2,5,7,8‐tetramethyl chroman‐2‐carboxylic acid) (0, 12, 24, 36, 48, 60 μL) was used as a standard to determine the Trolox equivalent capacity of the tested samples. TAC of each sample was calculated using a standard curve.

### 2.7 Evaluation of DNA Damages

Genomic DNA was isolated from HaCaT, HFF1 and SSCs cultured for 72 h in the above‐described conditions using the Genomic DNA Purification Kit (Life Technologies, Carlsbad, CA, USA). The pellet was dissolved in LTE buffer and the DNA concentration was determined using the NanoDrop One/OneC Microvolume UV–Vis spectrophotometer (Thermo Fisher Scientific, Grand Island, NY, USA). The DNA damage Assay Kit (AP sites, Colorimetric) (Abcam, Cambridge, UK) was used to monitor the formation of apurinic/apyrimidinic (AP) sites in each sample. The APR (Aldehyde Reactive Probe) reacts specifically with an aldehyde group on the open ring form of AP sites. According to the manufacturer's instructions, the DNA binding solution was added to each well and Streptavidin‐Enzyme Conjugate incubated at 37°C for 1 h. After the addition of Substrate Solution, absorbance was read at 450 nm using a microplate reader (Akribis Scientific, Common Farm, Frog Ln, Knutsford WA16 0JG, Great Britain).

### 2.8 Comet Assay

The comet assay kit (Abcam, Cambridge, UK) was applied to detect the DNA damage of cells. HaCaT, HFF1 and SSCs were cultured for 72 h under the above‐described conditions. According to the manufacturer's instructions, 20 μL of mixed solution was dripped into the round hole of a six‐well comet slide and solidified at 4°C in the dark for 15 min. After solidification, the coverslip was soaked in the lysis solution buffer at 4°C overnight. The slides were then washed and put in the alkaline buffer for 30 min. After electrophoresis, the coverslip was immersed in cold neutralisation buffer at 4°C for 15  min to remove alkali and detergent. Subsequently, the coverslip was stained with PI staining solution for 10 min. Last, the tail moment and the severity of DNA damage were quantitatively evaluated by fluorescence microscope (TCS SP5, Leica, Nussloch, Germany) and quantified by an image analysis (ImageJ, version 1.8.0, National Institutes of Health, Bethesda, MD, USA).

### 2.9 Senescence‐Associated β‐Galactosidase Staining

β‐Galactosidase assay was used to evaluate cell senescence after exposure to UV light. HaCaT, HFF1 and SSCs were cultured for 72 h in the above‐described conditions and then stained with the Senescence‐associated (SA) β‐Galactosidase Staining Kit (Cell Signalling Technology, Euroclone, Milan Italy) according to the manufacturer's instructions. The number of blue positive cells was detected under an inverted microscope and analysed with image software analysis (ImageJ, version 1.8.0, National Institutes of Health, Bethesda, MD, USA).

### 2.10 Statistical Analysis

Statistical analysis was performed using GraphPad Prism 9.0 software (GraphPad, San Diego, CA, USA). The experiments were performed two times with three technical replicates for each treatment. Kruskal–Wallis rank sum, two‐way analysis‐of‐variance ANOVA tests with Tukey's correction and Wilcoxon signed‐rank test were used. *p* value < 0.05 was assumed to be statistically significant. We considered *p < 0.05, **p < 0.01, ***p < 0.001, ****p ≤ 0.0001.

## 3 Results

### 3.1 Cell Viability and Proliferation

**FIGURE 1:**
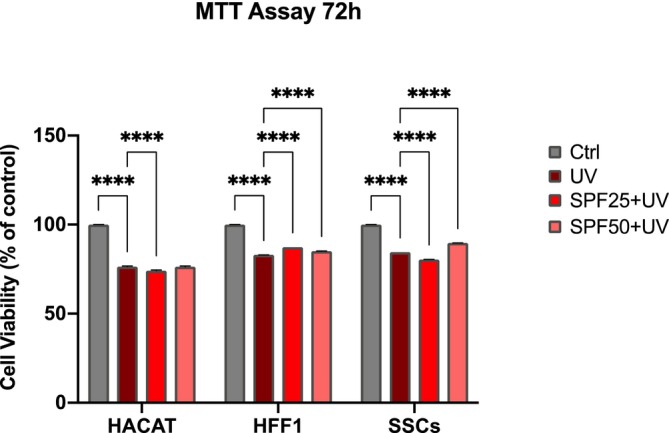
MTT Viability assay of HaCaT, HFF1 and SSCs after 72 h of pre‐treatment with creams and exposure to UV. Cell viability was expressed as % cell viability referred to untreated control cells. Data are expressed as mean ± SD (*n* = 6) referring to the control (****p ≤ 0.001).

**FIGURE 2:**
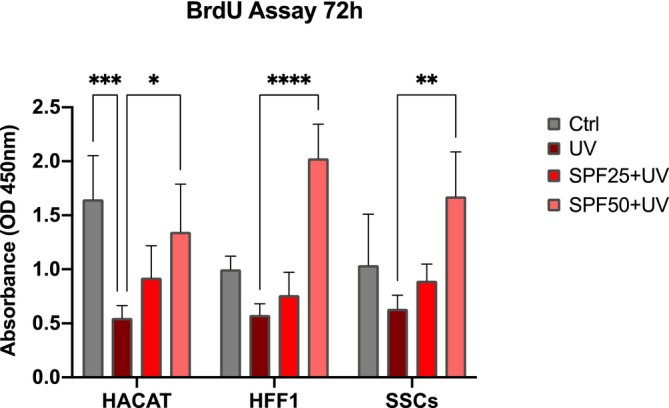
Proliferation of HaCaT, HFF1 and SSCs after 72 h of pre‐treatment with creams and exposure to UV. Cell proliferation is expressed in OD units as compared to control untreated cells. Data are expressed as mean ± SD (*n* = 6) referring to the control (*p ≤ 0.05; **p ≤ 0.01; ***p ≤ 0.001, ****p ≤ 0.001).

MTT cell viability (Figure 1) was performed after 72 h of pre‐treatment with SPF 25 or SPF 50+ creams (red and pink bars respectively) on HFF1, SSCs and HaCaT exposed or not to UV light (dark red and grey respectively). MTT analysis shows no significant difference between cells pretreated with the products (red and pink bars respectively) and untreated control cells (grey bar), while a significant difference was observed as compared to positive controls (UV) exposed to UV light without pre‐treatment with the creams. BrdU proliferation assays (Figure 2) was performed after 72 h of pre‐treatment with SPF 25 or SPF 50+ creams (red and pink bars respectively) on HFF1, SSCs and HaCaT exposed or not to UV light (dark red and grey respectively). Figure 2 showed the capability of the product in maintaining and enhancing cell proliferation even following exposure to oxidative stress. Both products (red and pink bars respectively) were effective in protecting cells from photo‐exposure by enhancing their response starting from the first hours of exposure, for all cell populations analysed.

### 3.2 Gene Expression Analysis

**FIGURE 3:**
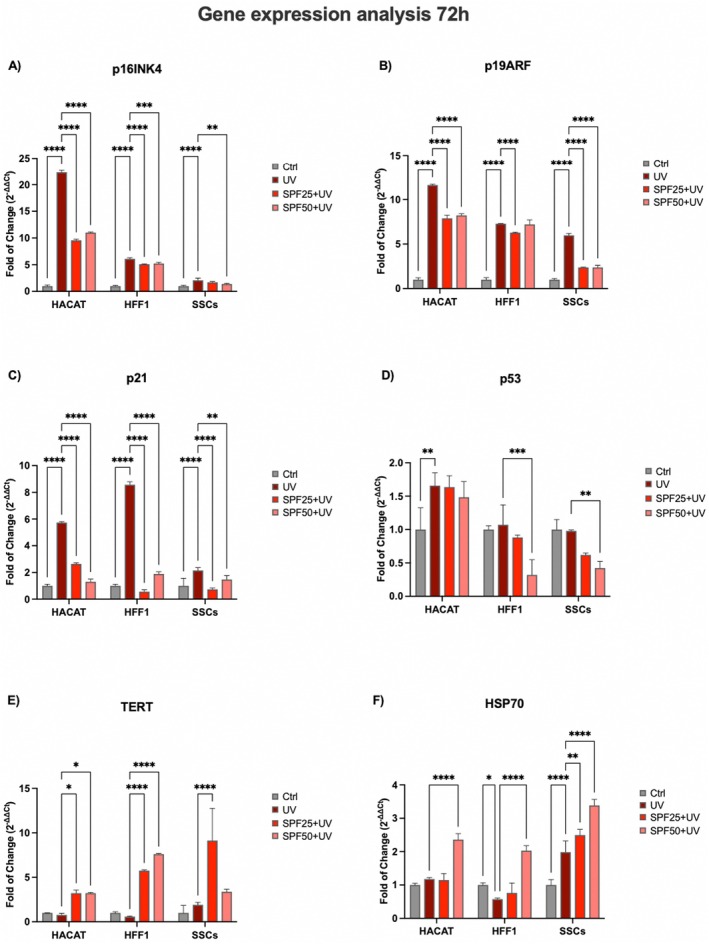
Expression of cell‐cycle regulator genes after 72 h of pre‐treatment with creams and exposure to UV. The expression of p16 (panel A), p19 (panel B) p21 (panel C), p53 (panel D), TERT (panel E) and HSP70 (panel F) was evaluated in HaCaT, HFF1 and SSCs after exposure to UV. The mRNA levels for each gene were normalised to Glyceraldehyde‐3‐Phosphate‐Dehydrogenase (GAPDH) and expressed as fold of change (2−ΔΔCt) of the mRNA levels observed in untreated control cells (Ctrl) defined as 1 (mean ± SD; *n* = 6). Data are expressed as mean ± SD referred to the control (*p ≤ 0.05), (**p ≤ 0.01), (***p ≤ 0.001), (****p ≤ 0.0001).

Gene expression analyses revealed that the key cell cycle regulators, p16, p19, p21 and p53 (Figure 3, panel A, B, C and D respectively) are upregulated in cells exposed to UV light (dark red bar) without creams pre‐treatment in all the cell populations tested. On the other hand, cells pretreated with the products (red and pink bars respectively) maintained a threshold of expression of p16, p19, p21 and p53 superimposable to that observed in untreated controls (grey bar) (Figure 3, panel A, B, C and D respectively). In cells exposed to UV light, the pre‐treatment with the creams was able to counteract cell senescence by reducing the expression levels of all the genes analysed (red and pink bars respectively), as compared to cells exposed to UV light not pre‐treated with creams (dark red bar). Moreover, cells cultured in the presence of the two creams, and exposed to UV (red and pink bars respectively), showed increased levels of TERT expression (Figure 3, panel E) as compared to the UV‐exposed positive control (dark red bar). At the same time, levels of HS70, the main Heat Shock Protein activated in response to stress, also show increased levels in treated cells (red and pink bars respectively), especially for SFF50+, as compared to untreated controls (grey bar), in all the conditions analysed (Figure 3, panel F).

### 3.3 Total Antioxidant Capacity

**FIGURE 4:**
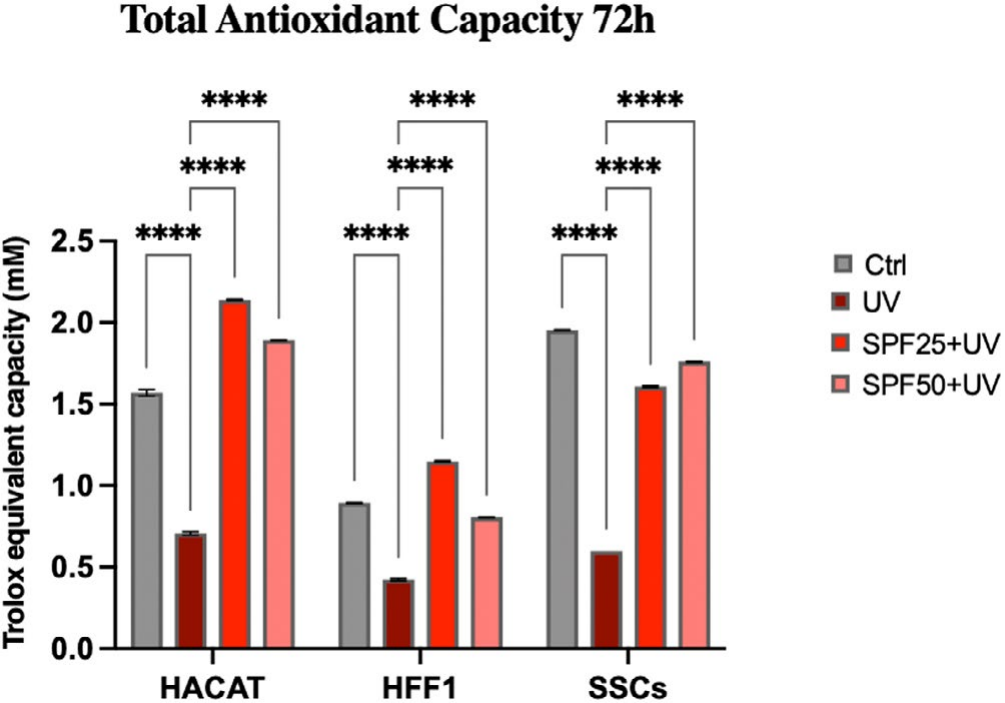
Total antioxidant capacity of HaCaT, HFF1 and SSCs after 72 h of pre‐treatment with creams and exposure to UV. Data are expressed as mean ± SD (*n* = 6, in triplicate with two experiments per group) (****p ≤ 0.001).

Figure 4 showed that cells undergoing pre‐treatment before exposure to oxidative stress (red and pink bars respectively) improved their total antioxidant capacity, as compared to untreated control cells (grey bar) and to cells exposed to UV light without cream pre‐treatment (dark red bar). These results were similar for all populations analysed.

### 3.4 Assessment of DNA Damages and Photolyase Activity

**FIGURE 5:**
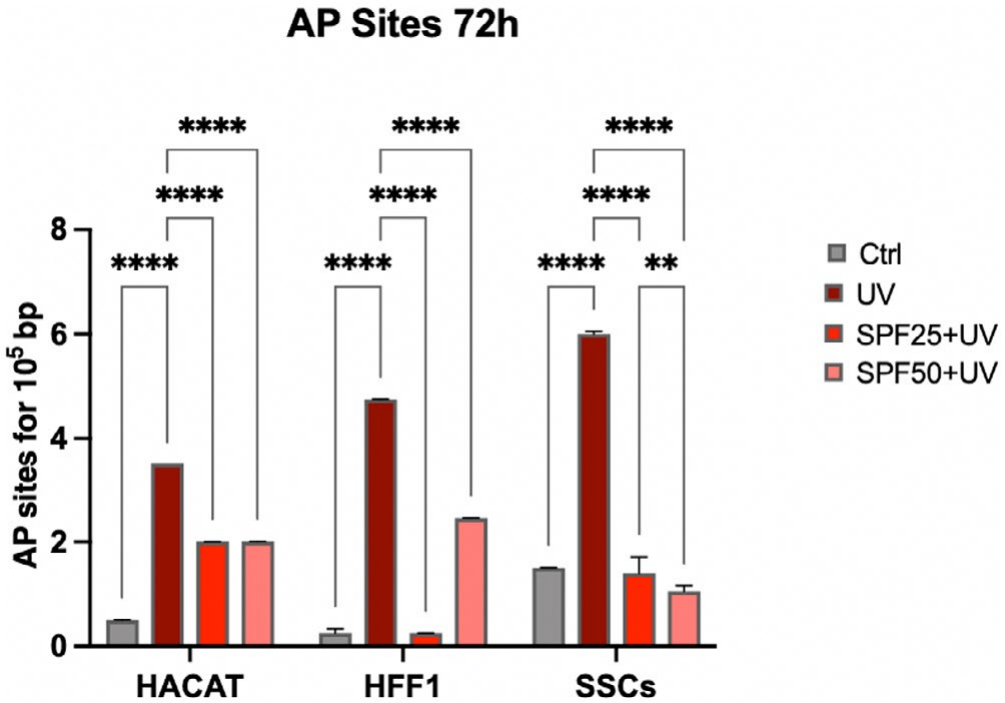
AP sites formation in HaCaT, HFF1 and SSCs after 72 h of pre‐treatment with creams and exposure to UV. Data are expressed as mean ± SD (*n* = 6, in triplicate with two experiments per group) (**p ≤ 0.01), (****p ≤ 0.0001).

Possible DNA damage induced by direct exposure to UV was then analysed. Interestingly, the presence of damages was reduced when cells were pretreated with the two creams before UV exposure (red and pink bars respectively), as compared to cells exposed to UV light without pre‐treatment (dark red bar) for all populations studied (Figure 5).

**FIGURE 6:**
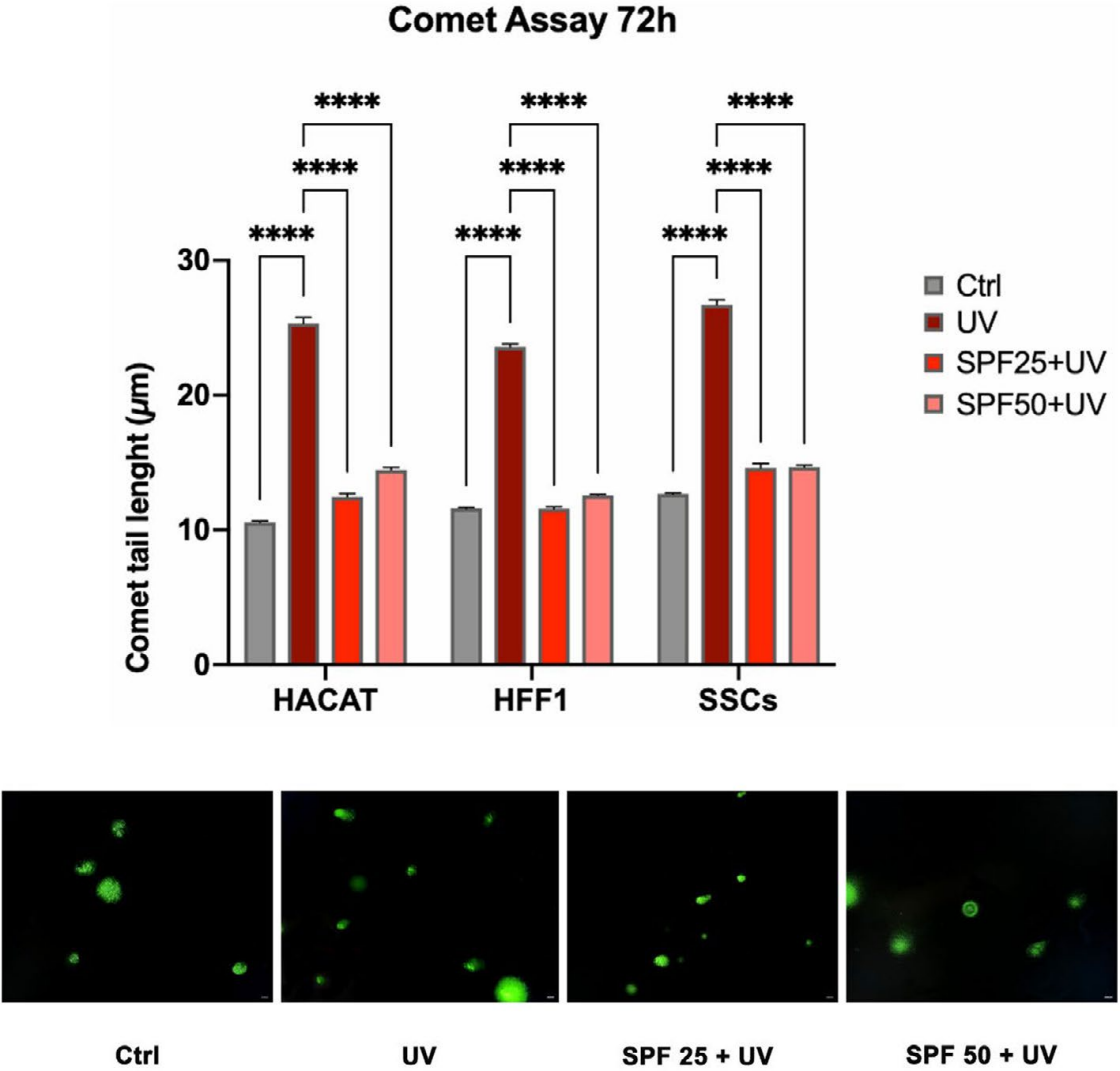
Comet assay in HaCaT, HFF1 and SSCs after 72 h of pre‐treatment with creams and exposure to UV. Data are expressed as mean ± SD (*n* = 6, in triplicate with two experiments per group) (****p ≤ 0.0001). The figures are representative of different independent experiments. Fields with the highest yield of positively stained cells are shown. Scale bars: 40 μm.

The Comet Assay was used to assess photolyase activity, which is involved in DNA repairing and detected by the formation of a comet visible in fluorescence. As shown in Figure 6, cells that were pretreated with the two creams for 72 h before UV exposure (red and pink bars respectively) showed a reduced comet tail length, as compared to cells that were exposed directly to the UV light (dark red bar).

### 3.5 β‐Galactosidase Assay

**FIGURE 7:**
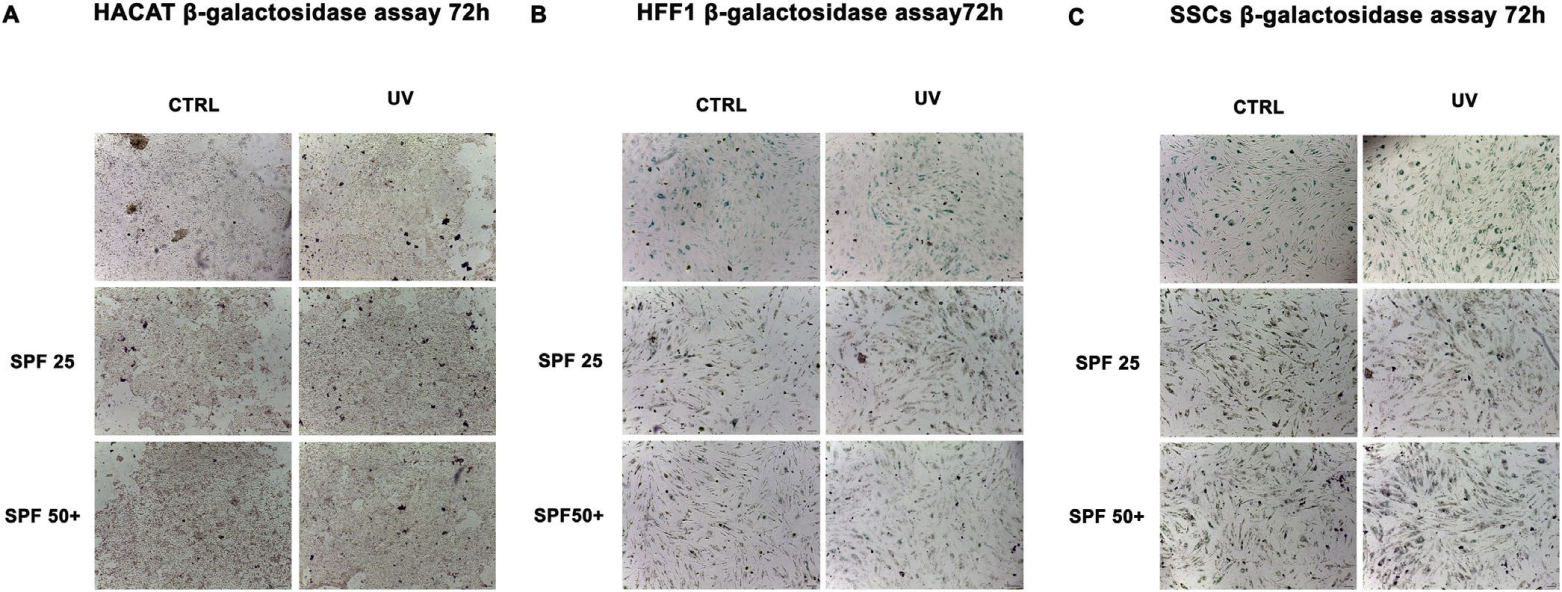
Senescence‐associated β‐galactosidase activity. β‐galactosidase was evaluated in HaCaT (panel A), HFF1 (panel B) and SSCs (panel C) after 72 h of pre‐treatment with creams and exposure to UV. All cells were compared to control untreated HSPCs (Ctrl). Scale bar = 100 μm.

Finally, β‐Galactosidase colorimetric assay was performed in cells that were pre‐treated with the cream before UV exposure (red and pink bars respectively). Figure 7 showed that creams can prevent and counteract premature senescence induced by UV light, as compared to untreated controls (grey bar). Indeed, the positive control cells (UV) showed some typical traits of aged cells, visible by the characteristic blue coloration, presenting a reduced proliferative and stress‐response capabilities.

## 4 Discussion

Ultraviolet radiation is a major cause of DNA damage in skin, mainly contributing to photoinduced skin aging [35]. Skin stem cells are innately very resistant to the aging process, being provided with very active DNA repair mechanisms. However, these mechanisms gradually become compromised along with aging, and the cells become more vulnerable to recurring UV exposure [36, 37]. The skin has endogenous photosensitizers able to absorb photons from the sun that trigger photo‐oxidation processes, resulting in ROS production [38]. Continuous and uncontrolled exposure to UVA rays can induce increased production of ROS and nitric oxide (NO), due to reduced catalase enzyme activity and protein kinase C (PKC) expression [39]. UVB exposure stimulates proinflammatory markers, including TNF‐α, IL‐6, iNOS and COX‐2, and can induce direct damage to cellular DNA through the formation of thymine dimers [40]. Damaged DNA can cause mutations or chromosomal rearrangements if not properly repaired, adversely affecting self‐renewal and stem cell potency, promoting skin aging and/or tumour formation [41, 42]. Within this context, we pre‐treated SSCs, HFF1 and HaCaT, with SPF 25 or SPF 50+ cream before exposure to UV light. The pretreatment was able to protect cells from premature senescence, reducing the number of blue positive senescent cells, as shown by β‐gal assay (Figure 7), and at the same time, increasing the expression of TERT (Figure 3, panel E). TERT is normally expressed in actively proliferating cells, preventing telomere shortening at each replicative cycle, an event typical of cells undergoing senescence [43]. Cells undergoing pretreatment did not show a decrease in proliferation rate after exposure to UV light, as compared to UV‐exposed cells not pretreated with the cream (positive control) (Figure 2). Also, gene expression analyses revealed that the two creams were able to maintain cell viability and proliferation despite exposure to strong oxidative stress, as the direct exposure to UV lamp (Figure 3). Cell cycle regulators, p16, p19, p21 and p53 were significantly downregulated in cells pre‐treated with the creams, as compared to cells exposed to UV light without pretreatment. All these markers are widely known to be upregulated in aging and senescence, originally identified as cell cycle inhibitors [44]. Moreover, upregulation of p19ARF followed by p53 and p21 results in triggering senescence and senescence‐associated secretory phenotype (SASP) [45]. On the other hand, we observed a significant upregulation of HSP70, which is normally released in response to environmental changes, to reduce cellular damage and activate repairing mechanisms [46, 47]. One of the primary damages caused by UV radiation is its impact on proteins, where it can break chemical bonds, leading to the unfolding or denaturation of these molecules. This can lead to loss of function or even cell death [27]. The unfolded protein response (UPR) is a stress response mechanism critical for cell survival. Hsp70 acts as a chaperone, maintaining the integrity of proteins within the cell [48]. In fact, under stress and non‐stress conditions, Hsp70 prevents the aggregation of misfolded proteins and coordinates their folding, allowing them to return to their native state [48, 49]. The increased expression level of HSP70 observed in cells pre‐treated with the creams (Figure 3, panel F) seems to exert a central role in protecting cells against UV light. To prevent photoaging and skin cancers and reduce exposure to DNA‐damaging agents (as UVR), the use of protective mechanisms preventing premature aging is necessary [50]. Using topical products enriched in phytochemicals and repairing enzymes could help in decreasing the number of DNA alterations by activating its corrective mechanisms [51]. The formation of apurinic/apyrimidinic (AP) sites is one of the prevalent lesions of DNA damaged by ultraviolet radiation [52]. These sites are formed in DNA by spontaneous loss of bases as in purification, by oxidation or by the action of DNA glycosylases [53]. Using an Aldehyde Reactive Probe (APR) that specifically reacts with an aldehyde group on the open ring of AP lesions, it is possible to quantify the DNA damage. Therefore, in cells pretreated with the two creams, we observed a reduced number of potentially damaged sites on DNA (Figure 5), mainly due to the implemented activity of the photolyase enzyme added to the formulation. The extracts are also able to decrease the tail length of the comet analysed, as reported in Figure 6, contributing to the activation of DNA repairing mechanisms. Exposure to ultraviolet rays is also responsible for the increased production of ROS [54]. ROS are spontaneously generated within cells in various subcellular organelles, especially in mitochondria and peroxisomes, during normal cellular metabolic reactions [55]. An adequate level of ROS is necessary for the normal physiological function of mammalian cells and, therefore, is essential for life [56]. Different biological molecules act as natural antioxidants to eliminate excessive ROS levels, mitigating oxidative stress and preserving cellular redox homeostasis [57]. The uncontrolled accumulation of ROS, on the other hand, turns out to be toxic, generating oxidative damage to biomolecules, including lipids, proteins, and DNA, impairing their ability to self‐renew, resulting in premature aging [38, 58]. Cells are highly self‐organising machines in which quality control triggering the production of macromolecules and organelles is balanced by the elimination of their aberrant counterparts [59]. The aging process is a progressive decline in cellular function, characterised by an increased susceptibility to tissue and organ disorders [60]. All signalling and quality control pathways are interconnected and contribute to biological resilience and the ability of an organism to restore homeostasis after a stressful event [61, 62]. Preventive interventions such as photoprotection and the use of products with antioxidant and reparative activity could be an important approach in maintaining cellular function, contributing to so‐called cellular rejuvenation [63]. The observed effects on different cell populations, especially on stem cells engaged in regenerating damaged tissue, allow cells to withstand a stressful event and UV‐induced senescence without the need for more invasive techniques, such as cellular reprogramming or epigenetic regulation. Currently, prevention and treatment strategies for photo‐aged skin focus mainly on strengthening the antioxidant defence of cells [64]. Several antioxidant phytochemicals, such as caffeic acid, ferulic acid, and gallic acid, ubiquitously present in plant‐based foods and beverages, have been shown to counteract oxidative stress in different skin populations [65, 66]. Moreover, when delivered in a controlled manner using nanodevices, their effect is further enhanced [67]. The presence of antioxidant molecules in the formulations of the two creams appears to enhance the response of cells to UV‐induced stress, improving their total antioxidant capacity in counteracting excessive ROS. The above‐cited biological activity of the products can also preserve cells from premature senescence and photo exposure damage (Figure 4). Natural extracts intended for topical use, which possess biological anti‐aging properties, have been proposed as potential enhancers of skin stem cell activation and regenerative capabilities, especially when combined with tissue engineering techniques. However, further research is necessary to thoroughly evaluate their effectiveness and safety in preventing and treating skin photoaging for clinical applications [68, 69].

## 5 Conclusions

In conclusion, photoprotection is the main weapon for reducing ultraviolet (UV) radiation‐induced skin damage and cancer. However, most of the products currently on the market lack molecules with scavenging and repairing activity for potential damage. Our findings demonstrate that creams formulated with specific sunscreens and phytochemicals effectively counteract the skin photoaging process induced by UV exposure. They achieve this by enhancing cell viability and proliferation, preventing premature cell senescence, boosting antioxidant activity, and reducing the risk of DNA damage while also facilitating the repair of damaged DNA. The results observed across all cell populations analysed indicate that the topical application of products containing repairing enzymes may significantly contribute to preventing UV‐induced damage, which can lead to abnormal phenotypic changes. The use of these innovative products, containing phytoplankton extract and the DNA Reactivator Complex, could represent an important breakthrough in the prevention of photo exposure damage.

## Author Contributions

**Sara Cruciani:** conceptualization (equal), formal analysis (equal), investigation (equal), methodology (equal), software (equal), writing – original draft (equal). **Giuseppe Garroni:** methodology (equal). **Diletta Serra:** methodology (equal). **Fikriye Fulya Kavak:** methodology (equal). **Rosanna Satta:** methodology (equal). **Fernanda Martini:** methodology (equal). **Mauro Tognon:** methodology (equal). **Carlo Ventura:** visualization (equal), writing – review and editing (equal). **Margherita Maioli:** conceptualization (equal), investigation (equal), supervision (equal), writing – review and editing (equal).

## Disclosure

The statements, opinions and data contained in all publications are solely those of the individual author(s) and contributor(s) and not of MDPI and/or the editor(s). MDPI and/or the editor(s) disclaim responsibility for any injury to people or property resulting from any ideas, methods, instructions, or products referred to in the content.

## Ethics Statement

The study was conducted in accordance with the Declaration of Helsinki and approved by an Ethical Committee (Clearance N. 0021565/2018, 22/03/2018‐Commissione Etica CNR).

## Consent

Informed consent was obtained from all patients in accordance with the EU and Italian ethical and medical regulations (Ethical Clearance N. 0021565/2018, 22/03/2018‐Commissione Etica CNR).

## Conflicts of Interest

The authors declare no conflicts of interest.

## Data Availability

The data of the current study are available inside the manuscript.
