# Correction to ‘Counteracting Skin Aging In Vitro by Phytochemicals’

**DOI:** 10.1111/jcmm.71114

**Published:** 2026-03-25

Sara Cruciani, Giuseppe Garroni, Diletta Serra, Fikriye Fulya Kavak, Rosanna Satta, Fernanda Martini, Mauro Tognon, Carlo Ventura, Margherita Maiol. “Counteracting Skin Aging In Vitro by Phytochemicals”, *Journal of Cellular and Molecular Medicine* 29, no. 7 (2025): e70530, https://doi.org/10.1111/jcmm.70530.

In the published article, an error was identified in Figure 7C in the Results section, in which an incorrect image was included in the panel labelled SPF25‐UV. The corrected Figure 7C is provided here, together with the corrected complete Figure 7. This correction does not affect the results or conclusions of the article.

Corrected Panel C

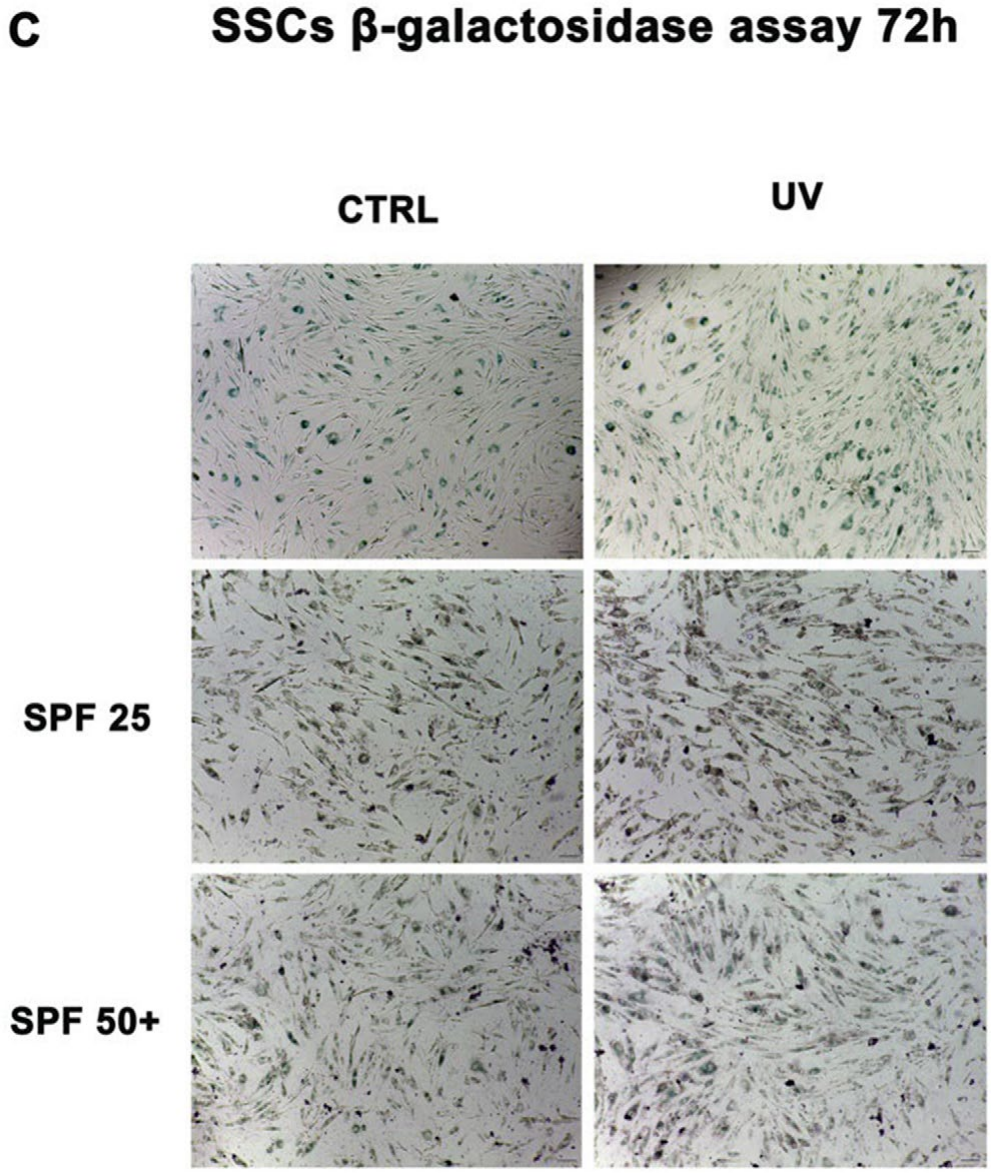

Corrected Figure 7

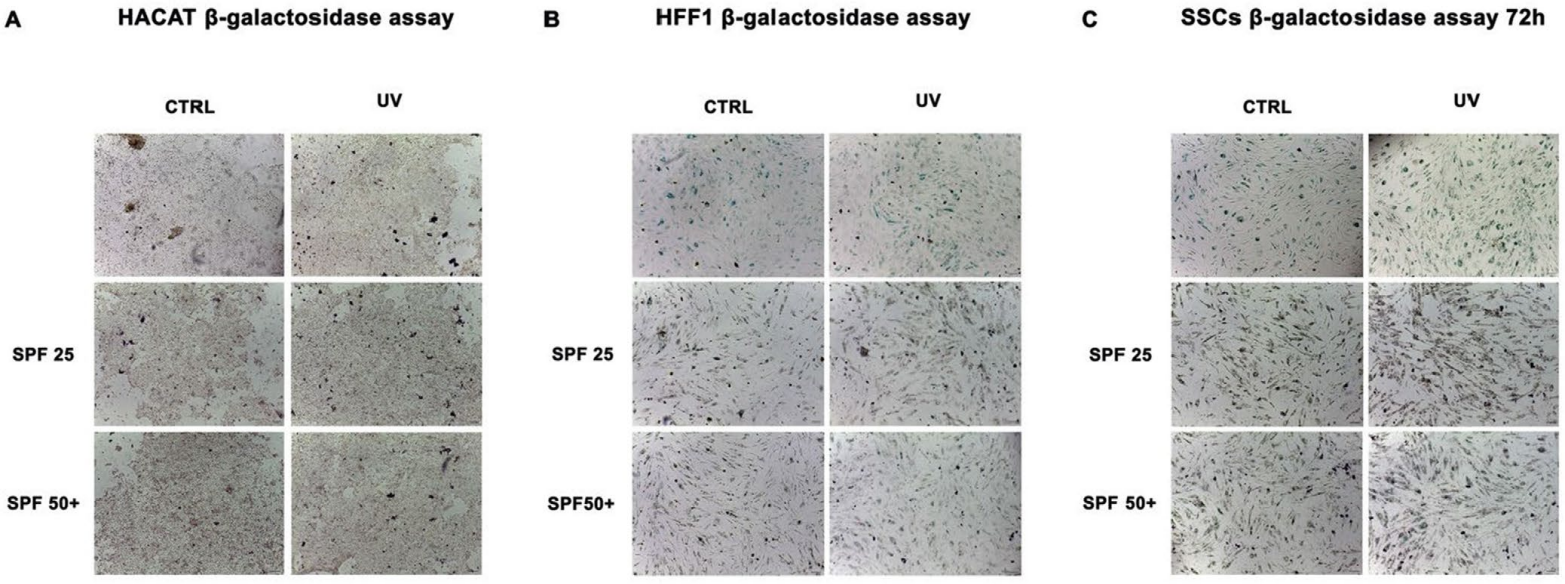

The authors apologise for this error.

